# Does HPV 16/18 infection affect p53 expression in invasive ductal carcinoma? An experimental study

**DOI:** 10.12669/pjms.304.4534

**Published:** 2014

**Authors:** Liang Hong, Shujie Tang

**Affiliations:** 1 Liang Hong, MD, Department of General surgery, Tianjin 4th Centre Hospital, Tianjin, 300140, China.; 2 Shujie Tang, MD, PhD, Department of Traditional Chinese Medicine, Medical School, Jinan University, Guangzhou, 510632, China.

**Keywords:** Invasive ductal carcinoma, HPV16/18, p53, Immunohistochemistry, PCR

## Abstract

***Objective*** **:** To determine the relations between human papilloma viruses type 16 and type 18 infection and the expression of p53 protein in invasive ductal carcinoma.

***Methods*** **:** We detected the expression of HPV 16/18 DNA and p53 protein in invasive ductal carcinoma in 45 cases, breast fibroadenoma in 20 cases and normal breast tissues in 20 cases. HPV detection was performed on paraffin sections using biotin-labeled probes by in situ hybridization and p53 protein expression was evaluated by immunohistochemistry.

***Results*** **:** The expression rate of HPV 16/18 DNA and p53 protein in invasive ductal carcinoma is significantly higher than those in breast fibroadenoma and normal breast tissues (p<0.05); the expression in cases with axillary lymph node metastasis is dramatically higher than those without (p<0.05); the expression of p53 protein increases with TMN staging advance. The expression of HPV16/18 DNA was significantly correlated with the expression of p53 protein (*p*<0.05).

***Conclusion*** **:** Both HPV16/18 infection and p53 mutation participate the occurrence and progress of invasive ductal carcinoma, and HPV 16/18 infection may be the major factor to cause p53 mutation.

## INTRODUCTION

Female breast cancer is one of the most prevalent malignancies all over the world.^1^ Recent studies have suggested that human papilloma virus (HPV) may be involved in the pathogenesis and progress of breast cancer.^2^ In addition, as an important tumor- suppressor gene, p53 and its production are essential to gene correction and chromosome stability.^3^ Some studies demonstrate p53 protein is the diagnostic index to primary breast cancer.^4^ However, up to now, few studies have been published in English literatures on the correlation between human papilloma virus type 16 and 18 (HPV 16/18) and p53 protein in breast cancer.

We detected the express of HPV16/18 DNA and p53 protein in 85 in-patients in the department of breast surgery of our hospital from September 2005 to October 2011, the purpose of our study was 1) To compare the expression of HPV 16/18 DNA and p53 protein in invasive ductal carcinoma, breast fibroadenoma and normal breast speciman; 2) To determine the correlation between HPV 16/18 infection and p53 gene in breast cancer to facilitate the clinical diagnosis.

## METHODS

***General Information: ***The specimens included invasive ductal carcinoma in 45 cases, breast fibroadenoma in 20 cases and normal breast tissues in 20 cases. The average age of cases was 41.5 years old (range from 23 to 58 years old) and according to the TNM staging of international union against cancer in 1997^4^, there was 11 cases with stage I, 25 with stage II and 9 with stage III; 29 cases with axillary lymph node metastasis and 16 cases without; all cases were diagnosed by intraoperative frozen section and regular postoperative pathological examination, and no case underwent chemotherapy, radiotherapy or immunotherapy before surgery. The study was carried out with the approval from the Ethics Committee of our Hospital and informed consent was obtained from all subjects.

***DNA Isolation*** ***: ***All samples were mounted in paraffin. The thickness of tissue section was 5μm, 3-5 sections were put into an EP tube, 1ml xylene was added and mixed up, and kept at room temperature for 30min, then spun down and the supernatant was removed, the process was repeated for three times, then the samples were kept at room temperature overnight; The sample was dipped in 100% Ethanol and xylene each for 30min two times, then dried into powder using 37ºC oven, and DNA extracting solution 50ul was added, heated overnight using 55ºC water baths, then boiled for 10min, after centrifuge, the supernatant was transferred into reaction tube, mixed up and centrifuged, sterilized de-ionized water as negative controls, positive controls supplied by the commercial kit.

 ***Detection of HPV 16/18 DNA:*** The HPV detect kit is produced by Beijing Jinqiao Biotechnology Co. LtD, and the sequence of HPV16/18 primer is 5′-GCAAGCAACAGTTACTGCGA-3′ and 5′-CAACAAGACATACATCGA CC-3′, and the amplified fragment length is 195bp. DNA Marker is from Takara company and PCR Thermal Cycler (type 9600) is from PE company (USA). PCR reaction condition: initial denaturation at 93°C for 5 min, followed by 30 cycles of 93°C for 30s, 55°C for30s , 72°C for 30s and a final extension at 72°C for 5 min. Analysis of PCR products: apply 10μl of PCR products into 1.5% agarose gel for electrophoresis, then stained by ethidium bromide, and compare the orange bands with the positive control bands at the same position under UV light.

**Fig.1:**
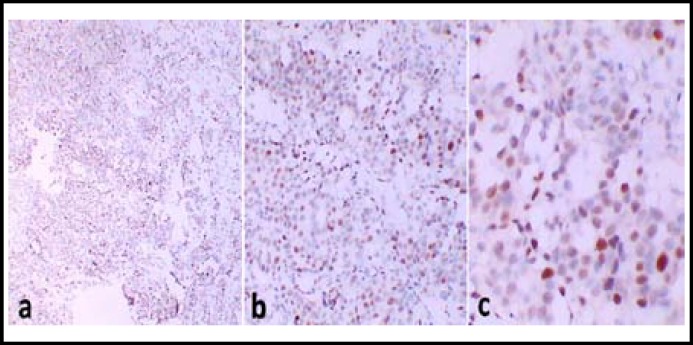
Immunostaining shows a clear and wide positive staining of p53 in a case of invasive ductal carcinoma. Original magnifications: ×40 (a), x 100(b) and x 200(c).

***Immunohistochemistry Staining and Result Analysis:*** Using immunohistochemical methods of SP: dewax the sections in water; dip the sections in 0.3% H_2_O_2 _and Methanol for 10-20min, respectively; rinse in water; antigen recover; PBS wash for three times, 1min for each time; serum incubation for 10 min; remove serum, add primary antibody incubate for 30-60min; PBS washing for 3 times, 2 min for each time; add secondary antibody incubate for 20 min, PBS washing 3 times, 2 min for each time; add SP compound incubate for 20-30 min; PBS washing 3 times, 2min for each time; DAB-H_2_O_2 _incubate 5-10 min; PBS wash and water wash; Harris Hematoxylin counter stain 5-10min; Wash back ground, dehydration, and mount. Result evaluation: the positive cell was defined by the brown-yellow particle of p53 protein occurs in nucleus and with regard to dyeing strength and positive range, the express in of the p53 was evaluated. Dyeing strength: no staining or staining similar with background is 0 point, lighter staining but darker than background is 1 point, and moderate staining and obviously higher than background is 2 points, strong staining with dark brown color is 3 points; Positive range: <10% is 0 point, 10%~24% is 1point, 25%~49% is 2 point, 50%~75% is 3 point, >75% is 4 point (Fig.1). Dyeing strength score plus Positive range score is the total scores, if it ≥ 2 points, then it is positive.

***Statistics analysis:*** Statistical analysis was performed using SPSS17.0 (SPSS Inc., Chicago, IL, USA). Descriptive analysis was performed by Chi-Square test. The correlation between HPV16/18 and p53 was determined with univariate logistic regression analysis. A probability value of < 0.05 was considered to indicate statistical significance.

## RESULTS

The expression rate of HPV 16/18 DNA and p53 protein in invasive ductal carcinoma (51.1% and 46.7%) is significantly higher than those in breast fibroadenoma (15.0% and 10.0%) and normal breast tissues (5.0% and 0%)(p<0.05); there is no significant difference between the two latter groups (p>0.05) (Table-I).

In the specimens of 45 invasive ductal carcinoma, the expression rate of HPV16/18 DNA and p53 protein in patients with axillary lymph node metastasis is significantly higher than those without (p<0.05); The expression rate of p53 protein increase with TMN staging advancing (p<0.05), while neither of the expression rates of HPV16/18 DNA and p53 protein is significantly correlated with age or tumor size (p>0.05) (Table-II).

In specimen of invasive ductal carcinoma, the positive correlation of HPV16/18 DNA with p53 protein expression (X^2^=6. 517, *P*<0.05) was found in the current study (Table-III).

## DISCUSSION

In the current study, we investigated the express of HPV16/18 DNA and p53 protein in invasive ductal carcinoma, breast fibroadenoma and normal breast tissues, to determine the correlation between HPV 16/18 infection and p53 gene in breast cancer. To the best of our knowledge, few studies have been published in English literatures on the issues.

**Table-I:** The expression of HPV16/ 18 DNA and p53 protein in breast cancer, breast fibroadenoma and normal breast tissues

***Group***	***n***	***HPV16/ 18 DNA ***			***p53 protein***	
		***positive***	***(%)***		***positive***	***(%)***
Normal breast tissue	20	1	5. 0		0	0
Breast fibroadenoma	20	3	15. 0●		2	10. 0●
Invasive ductal carcinoma	45	23	51. 1♦		21	46. 7♦

Compared to Normal breast tissue and breast fibroadenoma group: X^2^=12. 641, X^2^=7. 523,

X^2^=13. 788, X^2^=10. 736,

♦ *P*< 0. 01; Compared to normal tissues: X^2^=0. 598, X^2^=0. 526,

● *P*> 0. 05.

**Table-II:** The correlation of HPV16/18 DNA, p53 protein and breast cancer

**Classification**	**n**	**HPV16/18 DNA**			**p53 protein**	
		** Positive (%)**			** Positive (%)**	
***Age***						
< 50	16	7(43. 8)			6(37. 5)	
≥50	29	16(55. 2)			15(51. 7)	
X^2^		0. 538			0. 838	
*P*		0. 463			0. 360	
***Tumor Size***						
<2cm	18	8(44. 4)			7(38. 9)	
≥2cm	27	15(55. 6)			14(51. 9)	
X^2^		0. 534			0. 729	
*P*		0. 465			0. 393	
***TMN Stage***						
Stage I	11	4(45. 5)			3(27. 3)	
Stage II	25	13(48. 0)			10(40. 0)	
Stage III	9	6(66. 7)			8(88. 9)	
X^2^		1. 128			8. 555	
*P*		0. 569			0. 014	
***Lymph Nodes Metastasis***						
No	16	5(31. 3)			4(25. 0)	
Yes	29	18(62. 1)			17(58. 6)	
X^2^		3. 919			0. 683	
*P*		0. 048			0. 030	

**Table-III:** The correlation between HPV16/18 DNA and p53 protein

**HPV16/18 DNA**	**n**	**p53 protein**		**X** ^2^	***P***
		**Positive**	**Negative**		
Positive	23	14	9	6. 505	0. 011
Negative	22	7	15		

HPV is a non-enveloped DNA virus in the family of papilloma viruses. Band^5^ found HPV can immortalize normal breast cells and suggested its possible correlation to the occurrence of breast cancer for the first time. p53 is one kind of cancer-suppressor genes and correlated to some important functions, such as cell cycle control, DNA repair, cell differentiation and apoptosis, and p53 mutation occurred in almost 50% of human tumors.^6^

In the current study, the expression of HPV16/18 DNA and p53 protein in invasive ductal carcinoma is significantly higher than those in breast fibroadenoma and normal breast tissue, indicating that HPV16/18 infection is close correlated to the occurrence of breast cancer, and p53 mutation may play an important role in the occurrence of breast cancer. In addition, we found the expression of HPV16/18 DNA and p53 protein in patients withaxillary lymph node metastasis is significantly higher than those without, and the expression of p53 protein increase with TMN staging advancing, which demonstrate that the expression of HPV16/18 DNA and p53 protein may correlate closely with the prognosis of breast cancer. In this regard González-Sistal reported similar conclusion in his study. However, González-Sistal found the tumor size has significant correlation with the expression of p53,^7^ but we got different result and didn’t find the same significance, which may be contributed to the relatively small sample size in the current study.

Moreover, the present study demonstrated HPV16/18 DNA was closely correlated to the expression of p53 protein in invasive ductal carcinoma, indicating HPV infection and p53 mutation may cooperate in the occurrence and advance of breast cancer, and HPV infection may be the important factor to promote p53 mutation. In a clinical study, Yu foundp53 mutation and HPV 16/18 infection might coordinate in the development of lung squamous cell carcinomas.^8^ The current study is consistent with Yu’s conclusion. Although the two studies focused on different tumors, they demonstrated the close correlation between HPV16/18 and p53 in the development of different cancers.

P53 is activator of gene transcription process, which repress G1 phase, cell apoptosis and DNA repairation in cell cycle. Under normal circumstances, p53 expression is relatively low, but when DNA is injured, the expression of downstream gene such as p21, mdm-2, bax may increase and play an antiproliferative role under the induction of p53. E6 protein can combine p53 protein specifically and promote its rapid degradation, and result in the disorder of cell cycle, which is equivalent to p53 mutation, and the process is the classic ubiquitination degradation of p53 mediated by E6-AP. E6-AP is located around nucleus and p53 protein degradation occur in the same site. The combination of E6 and p53 may be mediated by E6-AP and its antisense target interactions can improve the expression of p53 in HPV infection cells. The C-terminal and N-terminal of E6 play an important role when interacting with p53 and promoting p53 degradation, among which, C-terminal is important to the combination with p53 and N-terminal is necessary to the degradation of p53.^9^^,^^10^ In addition, E6 may bind the transcription factor CBP/P300 by non-dependent pathway of p53, reduce the activation effect of p53 on downstream molecule, making the cells across the G1/S checkpoint into S phase.^11^ Consequently, HPV 16/18 infection and p53 mutation as well as their cooperation may trigger a series of molecular events and lead to cell immortalization or transformation.

In conclusion, the current study demonstrated the HPV infection and p53 mutation participate the occurrence and advance of breast cancer, and HPV infection may be an important factor to promote p53 mutation. HPV16/18 DNA and p53 protein can work as the molecular marker to facilitate the treatment and diagnosis of breast cancer. However, the current study has its limitations and some detailed molecular mechanism about these points was not studied clearly as such more research need to be performed in the future.
